# *Enterococcus faecium* Mediastinitis Complicated by Disseminated *Candida parapsilosis* Infection after Congenital Heart Surgery in a 4-Week-Old Baby

**DOI:** 10.1155/2015/543685

**Published:** 2015-10-28

**Authors:** Hanna Renk, Felix Neunhoeffer, Florian Hölzl, Michael Hofbeck, Matthias Kumpf

**Affiliations:** ^1^University Children's Hospital Tübingen, Department of Pediatric Cardiology, Pulmonology and Intensive Care Medicine, Hoppe-Seyler-Straße 1, 72076 Tübingen, Germany; ^2^Institute of Medical Microbiology and Hygiene, University of Tübingen, Elfriede-Aulhorn-Straße 6, 72076 Tübingen, Germany

## Abstract

*Background*. Cardiac surgery offers multiple treatment options for children with congenital heart defects. However, infectious complications still remain a major cause of morbidity and mortality in these patients. Mediastinitis is a detrimental complication in children undergoing cardiac surgery. The risk of mediastinitis after delayed sternal closure is up to 10%. *Case Presentation*. We report a case of *Enterococcus faecium* mediastinitis in a 4-week-old female baby on extracorporeal membrane oxygenation after Norwood procedure. Although repeated antibiotic irrigation, debridement, and aggressive antibiotic treatment were started early, the pulmonary situation deteriorated. *Candida parapsilosis* was isolated from bronchoalveolar lavage after pulmonary hemorrhage. Disseminated *C. parapsilosis* infection with pulmonary involvement was treated with liposomal amphotericin B. Subsequently, inflammatory markers increased again and eventually *C. parapsilosis* was isolated from the central venous catheter. *Conclusion*. Children undergoing delayed sternal closure have a higher risk of mediastinitis. Therefore, antibiotic prophylaxis, for example, for soft tissue infection seems justified. However, long-term antibiotic treatment is a risk factor for fungal superinfection. Antifungal treatment of disseminated *C. parapsilosis* infection may fail in PICU patients with nonbiological material in place due to capacity of this species to form biofilms on medical devices. Immediate removal of central venous catheters and other nonbiological material is life-saving in these patients.

## 1. Introduction

Mediastinitis is a rare but detrimental complication of sternotomy, present in 0.2–2% of all cardiac surgeries [1]. Several treatment procedures have been established and include revision with open dressings, closed irrigation, reconstruction with soft tissue flaps, and, recently, vacuum assisted closure (VAC). However, there is no consensus regarding the best surgical treatment strategy and the duration of antibiotic therapy for severe mediastinal infection. Long-term antibiotic treatment for tissue infection in Pediatric Intensive Care Unit (PICU) patients is a risk factor for fungal superinfection [2].

## 2. Case Presentation

A four-week, full-term baby girl presented to PICU after Norwood procedure for hypoplastic left heart syndrome, unbalanced atrioventricular septal defect, and hypoplastic aortic arch. Heart failure was present and the risk of immediate anatomic correction considered too high. Therefore, pulmonary artery banding was the primary cardiac surgery. Subsequently, the Norwood procedure with augmentation of the aortic arch was performed and a modified Blalock-Taussig shunt was set up using prosthetic material. Low cardiac output required the implementation of venoarterial extracorporeal membrane oxygenation (vaECMO) with open chest and silicon sheeting. Mediastinal tissue appeared inflamed during the procedure and a swab was taken under sterile conditions.* Enterococcus faecium* was isolated and cefazolin prophylaxis was changed to treatment of mediastinitis with vancomycin and gentamicin, according to the resistogram.

The open chest was extensively irrigated with nebacetin (bacitracin/neomycin) on days three, five, and six. Drains were flushed, hematomas were removed, and mediastinal tissue and wound margins were vitalized. Wound swabs were taken and a sterile wound cover was applied. The third irrigation was followed by vaECMO weaning, application of a new wound sheeting, and sternal closure.* Enterococcus faecium* grew in all wound swabs and the vancomycin dose was increased to 60 mg/kg due to the severity of the infection and poor penetration into lung tissue [3]. Tigecycline was also added.

Four days after sternal closure, not only* Enterococcus faecium* but also* Candida parapsilosis* was cultured from urine and pleural drainage. C-reactive protein (CRP) peaked at 12 mg/dL and fluconazole (5 mg/kg/d) was started to prevent fungal superinfection. Finally, pulmonary hemorrhage and atelectasis of both upper pulmonary lobes evolved and mediastinal fluid was detected in a chest CT. High-frequency oscillation (HFO) with 100% O_2_ became necessary. Bronchoscopy was performed and* C. parapsilosis* was also isolated from BAL. The working diagnosis was mediastinitis due to* Enterococcus faecium* complicated by acute disseminated* C. parapsilosis* infection with pulmonary involvement. The clinical condition improved within the next five days, ventilation was switched to pressure control, and CRP dropped to 1 mg/dL.

**Figure 1:**
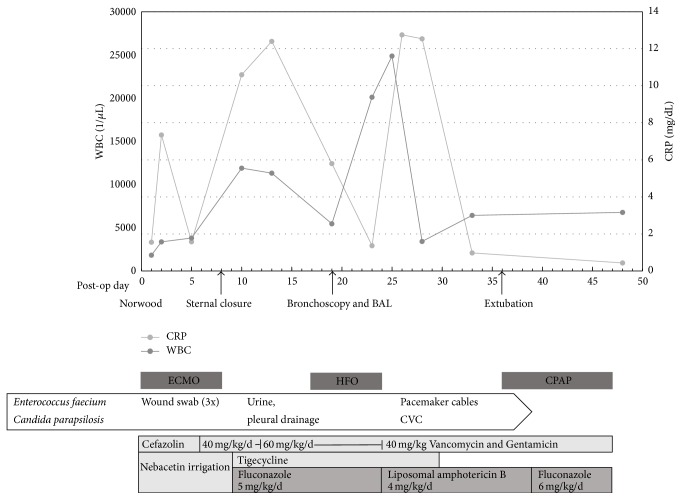
Hospital course. Serum C-reactive protein (CRP) level and leucocyte count (WBC) peaked after Norwood procedure, sternal closure, during pulmonary hemorrhage and invasive* C. parapsilosis* infection. Isolation of* Enterococcus faecium* and* C. parapsilosis* and mode and duration of treatment are shown. BAL: bronchoalveolar lavage, ECMO: duration of extracorporeal membrane oxygenation, HFO: duration of high-frequency oscillation, CPAP: duration of continuous positive airway pressure support, and CVC: central venous line.

Subsequently, however, CRP levels returned to 12 mg/dL within two days, and there was a marked leucocytosis of 25,000 cells/*μ*L (Figure 1). Resistance of* C. parapsilosis* against fluconazole was considered and antifungal treatment was changed from fluconazole to amphotericin B (4 mg/kg/d), while continuing the antibiotic regimen. However, this peak of inflammatory markers did not fit to the clinical improvement. The central venous catheter (CVC) was switched to an antibiotic-coated device and the pericardial pacemaker cables were removed.* C. parapsilosis* was isolated from the CVC and* Enterococcus faecium* from the pacemaker cables. Two weeks later,* C. parapsilosis* had cleared from the urine culture. Amphotericin B treatment was stopped and prophylactic fluconazole was given. Thirty-five days after cardiac surgery the baby was extubated and subsequently weaned from continuous positive airway pressure (CPAP). Duration of antibiotic treatment was three weeks of tigecycline and six weeks of vancomycin/gentamicin. Antifungal treatment had been given for a total of six weeks.

## 3. Discussion

Mediastinitis is a rare but worrying complication of congenital heart surgery with significant morbidity and mortality. This was a severe case of* Enterococcus faecium* mediastinitis complicated by disseminated* C. parapsilosis* infection with pulmonary involvement.

### 3.1. Surgical Management

Currently, there is no consensus regarding the best treatment strategy for postsurgical mediastinitis in children. VAC has promising results in adults and retrospective studies have also demonstrated its effectiveness in small children. By application of negative pressure to wound tissue, VAC enhances local blood perfusion and promotes formation of granulation tissue and approximation of wound edges. However, the negative pressure may compress cardiac cavities and subsequently impair diastolic filling of the ventricles and cardiac output [1]. In this case study the patient had decreased ventricular contractility and coagulation problems; the risk of hemodynamic deterioration with VAC was considered too high. Moreover, irrigation and extensive wound debridement seemed to be a safe method with favorable outcome. In the study of Ugaki et al., 2010, 20 children with mediastinitis were reviewed and continuous irrigation was used as first-line therapy followed by VAC for second-line therapy in cases where treatment had failed [4]. The outcome was favorable with no reinfections and low mortality. Therefore, it was inferred that repeated extensive irrigation with nebacetin and debridement combined with intensive antibiotic treatment would be an impervious procedure as long as our patient was on vaECMO. Although irrigation was stopped after sternal closure, it may be that continuous irrigation, as proposed in Ugaki's study, could have been a reasonable or even better option [4].

### 3.2. Antibiotic Management

Mediastinitis is mainly caused by intraoperative contamination, either endogenously from the patient, from the surgical team, or from the operating room air. Prolonged sternal closure increases the rate of postoperative infection [1]. Systemic antibiotic prophylaxis in cardiac surgery is questionable, since it may select resistant organisms. However, stagnant blood or hematomas in the mediastinum serve as an ideal medium for bacterial growth and, hence, preventive antibiotic therapy for the most common organisms seems reasonable and is accepted worldwide [1]. Currently, there are no studies that evaluate the optimal regimen or duration of antimicrobial treatment for mediastinitis in children. Common empiric regimens include vancomycin plus a third- or fourth-generation cephalosporin, carbapenems, or aminoglycosides. Timely identification of the organism facilitates effective antimicrobial treatment. In general, 3 to 8 weeks of antibiotic therapy is recommended, depending on the severity of the infection and possible sternal osteomyelitis [5]. In our case,* Enterococcus faecium* was the causative organism, and the combination of vancomycin and synergistic gentamicin was chosen. Penetration of vancomycin into tissue varies by site, and levels achieved in the lung are only about 25% of serum concentration [3]. To achieve appropriate lung concentration, the vancomycin dose was increased when the pulmonary situation deteriorated and tigecycline was added as a bacteriostatic agent with excellent skin-tissue penetration [6]. Tigecycline is highly active against* Enterococcus spp*. However, there are sparse data concerning the safety of treatment courses longer than two to three weeks [6].

### 3.3. Invasive* Candida* Infection (ICI)

This is a serious complication in PICU patients with an incidence of 3.5/1000 admissions [2]. It presents as either candidemia or disseminated candidiasis. Increasing rates of non-*Candida albicans* species have been reported in neonatal and pediatric populations and* C. parapsilosis* is the second most common isolate in European PICUs [7]. In children with congenital heart disease,* C. parapsilosis *was even more prevalent than* C. albicans* (isolated in 54% of all ICIs) [8].* C. parapsilosis* is the most frequent species in neonates and children <2 years, and transmission between patients or from healthcare personnel is common [7].* C. parapsilosis* seems less virulent than other non-*C. albicans* species, but it shows a selective preference to adhere onto plastic medical devices and readily forms biofilms. Therefore, the presence of CVCs increases the risk of* C. parapsilosis* infection and the high incidence is partly due to extensive use of indwelling catheters [7, 8]. Furthermore, parenteral nutrition provides a high glucose and lipid environment and enhances biofilm formation especially in this species [9]. Endotracheal intubation, duration of mechanical ventilation, and the presence of candiduria are other known risk factors for candidemia in general [7, 10].

Retrospectively, all risk factors for* C. parapsilosis* superinfection were present in our patient. Prolonged PICU stay, long duration of antibiotic therapy, age <2 years, candiduria, endotracheal intubation and mechanical ventilation, parenteral nutrition, and presence of a CVC for 25 days all favored disseminated candidemia. Disseminated infection may involve the lungs, liver, kidney, and brain. The clinical and laboratory signs of invasive or disseminated* C. parapsilosis* infection are unspecific and sepsis-like. Leucocytosis and thrombopenia, as well as elevation of liver enzymes, are often noticed [11]. PICU patients are at the highest risk of death due to candidemia (mortality rate 12-13%), but mortality due to* C. parapsilosis* does not differ from other species [2, 7, 11].

Antifungal treatment with the widespread use of azoles has partially influenced the shift towards higher percentages of non-*C. albicans* species and may promote the development of resistance [11]. However, in neonates, evidence for efficacy and pharmacokinetic data of fluconazole treatment are better compared to liposomal amphotericin B treatment [12]. Therefore, we choose fluconazole as a first-line agent. Although low, development of resistance of* C. parapsilosis* to azoles is possible and treatment was changed to liposomal amphotericin B, which is another possible agent for the treatment of ICI in neonates according to the ESCMID guideline [12]. Fortunately, after seven weeks, our patient was discharged from PICU.

## 4. Conclusion

Children on ECMO and delayed sternal closure have a higher risk of mediastinitis. Currently, there is no consensus regarding the optimal surgical treatment strategy and the duration of antibiotic therapy for severe mediastinal infection. Antibiotic prophylaxis, for example, for soft tissue infection seems justified in children on ECMO. However, long-term antibiotic treatment is a risk factor for fungal superinfection. The present case reports on a nosocomial* Enterococcus faecium* mediastinitis complicated by disseminated* C. parapsilosis* infection including colonization of the CVC.* C. parapsilosis* is known to form biofilms on nonbiological material. Antifungal treatment of disseminated* C. parapsilosis* infection may fail in PICU patients with nonbiological material in place due to capacity of this species to form biofilms on medical devices. Immediate removal of central venous catheters and other nonbiological material is life-saving in these patients.

## Abbreviations

BAL: Bronchoalveolar lavage
CPAP: Continuous positive airway pressure
CRP: C-reactive protein
CT: Computed Tomography
CVC: Central venous catheter
HFO: High-frequency oscillation
ICI: Invasive* Candida* infection
PICU: Pediatric Intensive Care Unit
VAC: Vacuum assisted closure
vaECMO: Venoarterial extracorporeal membrane oxygenation.

## References

[1] Durandy Y. (2010). Mediastinitis in pediatric cardiac surgery: prevention, diagnosis and treatment. *World Journal of Cardiology*.

[2] Zaoutis T. E., Prasad P. A., Localio A. R. (2010). Risk factors and predictors for candidemia in pediatric intensive care unit patients: implications for prevention. *Clinical Infectious Diseases*.

[3] Cruciani M., Gatti G., Lazzarini L. (1996). Penetration of vancomycin into human lung tissue. *Journal of Antimicrobial Chemotherapy*.

[4] Ugaki S., Kasahara S., Arai S., Takagaki M., Sano S. (2010). Combination of continuous irrigation and vacuum-assisted closure is effective for mediastinitis after cardiac surgery in small children. *Interactive Cardiovascular and Thoracic Surgery*.

[5] Long S. S., Pickering L. K., Prober C. G., Long S. S. (2009). Postoperative mediastinitis and sternal osteomyelitis. *Principles and Practice of Pediatric Infectious Diseases*.

[6] Ellis-Grosse E. J. E., Babinchak T., Dartois N., Rose G., Loh E. (2005). The efficacy and safety of tigecycline in the treatment of skin and skin-structure infections: results of 2 double-blind phase 3 comparison studies with vancomycin-aztreonam. *Clinical Infectious Diseases*.

[7] Dotis J., Prasad P. A., Zaoutis T., Roilides E. (2012). Epidemiology, risk factors and outcome of *Candida parapsilosis* bloodstream infection in children. *Pediatric Infectious Disease Journal*.

[8] San Miguel L. G., Cobo J., Otheo E. (2006). Candidemia in pediatric patients with congenital heart disease. *Diagnostic Microbiology and Infectious Disease*.

[9] Silva S., Negri M., Henriques M., Oliveira R., Williams D. W., Azeredo J. (2011). Adherence and biofilm formation of non-*Candida albicans Candida* species. *Trends in Microbiology*.

[10] MacDonald L., Baker C., Chenoweth C. (1998). Risk factors for candidemia in a children's hospital. *Clinical Infectious Diseases*.

[11] Jordan I., Hernandez L., Balaguer M. (2014). *C. albicans*, *C. parapsilosis* and *C. tropicalis* invasive infections in the PICU: clinical features, prognosis and mortality. *Revista Espanola de Quimioterapia*.

[12] Hope W. W., Castagnola E., Groll A. H. (2012). ESCMID^∗^ guideline for the diagnosis and management of *Candida* diseases 2012: prevention and management of invasive infections in neonates and children caused by *Candida* spp. *Clinical Microbiology and Infection*.

